# Depression prevalence estimates in Spain (1995–2021): a systematic review

**DOI:** 10.1515/med-2026-1498

**Published:** 2026-08-10

**Authors:** Sergio Ruiz-Sánchez, Aurora González-Teruel, Enric Novella

**Affiliations:** Departament d’Història de la Ciència i Documentació, Universitat de València, València, Spain; Institut d’Història de la Medicina i de la Ciència López Piñero, Universitat de València, València, Spain

**Keywords:** depression, prevalence, epidemiology, systematic review, Spain

## Abstract

**Introduction:**

This study aimed to systematically review and synthesise heterogeneous depression prevalence estimates in the Spanish general population and analyse their variation according to methodological factors over recent decades.

**Content:**

Searches were conducted in MEDLINE, Embase, Web of Science, Scopus, PsycINFO, ÍnDICEs CSIC, and PSICODOC. Inclusion criteria were: reporting incidence, prevalence, or mean depression scores; population ≥14 years; n ≥1,000; use of validated assessment methods. The review followed PRISMA 2020 guidelines. Study quality was assessed using the Joanna Briggs Institute tool. Findings were synthesised qualitatively. 13 studies were included; most were international (n=7), cross-sectional (n=11), and used diagnostic interviews (n=8). Comparable studies (n=11) showed an increase in prevalence estimates in recent years, ranging from approximately 4–22 %, depending on assessment method and study period. Several studies (n=7) reported significant associations with female gender and unfavourable sociodemographic conditions. Most studies (n=11) were high quality.

**Summary:**

Evidence derived from heterogeneous estimates suggests an increase in reported depression prevalence among comparable studies from 1995 to 2021. Despite methodological variation, women were affected twice as often as men, and unfavourable sociodemographic conditions were linked to higher risk. Early-pandemic increases likely reflect reactive psychological distress. This is the first systematic review on this topic.

**Outlook:**

Future research should use probability sampling and harmonised screening and diagnostic methods to improve representativeness and comparability.

## Introduction

Depression causes significant suffering for those affected and represents a major public health issue. On the one hand, it is a common reason for consultation in both primary care and specialised mental health services. On the other, it entails a high social and economic cost [1], 2].

According to data from the Global Burden of Disease Study (GBD), the World Health Organization (WHO) estimates that approximately 280 million people suffer from depression [3]. In 2021, depression ranked as the second leading global cause of Years Lived with Disability (YLDs) (56.3 million) and was the third fastest-growing cause of YLDs since 2010 (16.4 %), alongside diabetes (25.9 %) and anxiety disorders (16.7 %) [4]. Furthermore, in 2019, depressive disorders became the leading cause of disease burden among mental disorders, accounting for 37.3 % of all Disability-Adjusted Life Years (DALYs), ahead of anxiety disorders (22.9 %) and schizophrenia (12.2 %) [5]. It is also important to note the sociodemographic disparities observed in the epidemiology of depression, which affect women and individuals with disadvantaged socioeconomic conditions to a greater extent [6], 7].

Based on GBD data, Ge et al. [8] analysed trends in the incidence and disease burden of major depressive disorder (MDD) across 204 countries and territories between 1993 and 2017, and highlighted that Spain had the highest increase in both indicators, with an approximate rise of 30 %. This finding is consistent with a broader increase in demand for psychiatric care in Spain, as reflected in the number of referrals to psychiatry, which rose from 267,311 in 2013 to 729,019 in 2024 [9]. Likewise, the marked rise in the consumption of anxiolytics (175 %) and antidepressants (274 %) in recent decades is also noteworthy [10], [11], [12], [13], [14], with levels exceeding the average for the Organisation for Economic Co-operation and Development (OECD) [15], 16].

Against this background, Spain was more severely affected than other European countries by the 2008 global economic crisis, with unemployment rising to record levels (26.1 %) [17] and the implementation of austerity policies that steadily undermined the health system and social protection networks [18], [19], [20]. Similarly, the COVID-19 pandemic struck Spain with particular intensity, leading to the second largest decline in gross domestic product (GDP) in the European Union (EU) (−10.9 %) [21] and the highest excess mortality ratio during the first wave (80.8 %) [22]. In this regard, several factors such as economic hardship, job insecurity, reductions in health expenditure and a lack of social support have been consistently linked to poorer mental health outcomes and higher levels of depression, particularly among women and socio-economically disadvantaged groups [23], [24], [25], [26], [27], [28], [29].

Taken together, these data suggest a substantial and potentially increasing burden of depression in Spain. However, epidemiological studies of depression in Spain remain limited and highly heterogeneous in terms of populations, samples, assessment methods, study periods, and geographical coverage [2], [30], [31], [32], [33]. Furthermore, to date, only one systematic review on the prevalence of depression in Spain has been conducted [34], but it did not focus on the general population and presented important methodological limitations, including broad inclusion criteria, a limited search strategy, and the absence of a risk-of-bias assessment. As a result, it remains difficult not only to provide a consistent interpretation of depression prevalence estimates in Spain, but also to assess whether these estimates have changed over recent decades. This evidence gap also hampers the identification of factors that may be influencing reported estimates and potential trends, and limits the development of appropriate health strategies and policies.

Considering the above, we hypothesised that, among methodologically comparable general-population studies, depression prevalence estimates in Spain would tend to be higher in more recent than in earlier periods. We also expected these estimates to be consistently higher among women and individuals exposed to unfavourable sociodemographic conditions.

Accordingly, the main research question was: do available studies assessing depression prevalence in the Spanish general population indicate an increase in depression prevalence estimates over recent decades? To answer this question, the aim of this study was to systematically review and synthesise heterogeneous depression prevalence estimates reported in Spanish general-population studies and to analyse their variation according to methodological factors over recent decades. This review allowed for a comparison of the figures and trends observed in Spain with those reported in other countries, the evaluation of the impact of the COVID-19 pandemic, the description of the relationship between depression and various sociodemographic variables, and a brief analysis of contextual factors that may help interpret the trends observed in Spain.

## Methods

The systematic review was conducted in accordance with the PRISMA 2020 guidelines [35].

### Eligibility criteria

The condition under study was depression, the context was Spain, and the target population was the general population. Studies were included if they met the following criteria: I) reported incidence, prevalence, or mean scores of MDD, Persistent Depressive Disorder (PDD), or unspecified depression (UD); II) included the Spanish population aged ≥14 years; III) had a sample size of ≥1,000 participants; and IV) used validated psychometric instruments and/or internationally recognised standardised diagnostic criteria (e.g., Diagnostic and Statistical Manual of Mental Disorders –DSM– or International Statistical Classification of Diseases and Related Health Problems –ICD). A minimum sample size of 1,000 participants was set to ensure stable and precise prevalence estimates, in accordance with previous epidemiological and statistical literature [36], [37], [38], [39], [40], [41].

**Figure 1:**
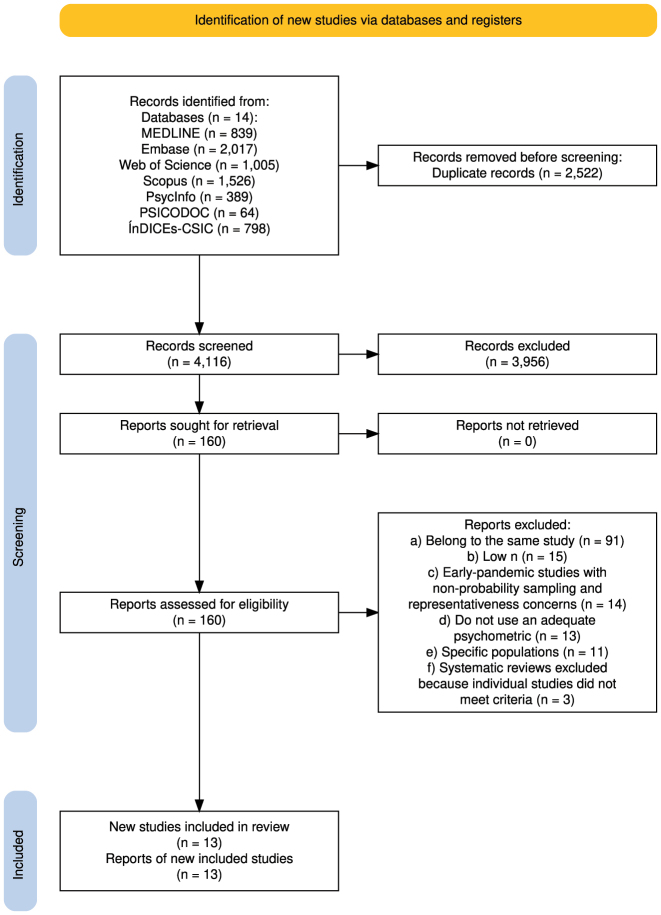
PRISMA flow diagram.

Reports not meeting the inclusion criteria were excluded. In addition, studies were excluded if their data were based on specific populations, defined as groups with characteristics likely to influence the prevalence of depression in a differential manner (e.g., older adults, students, workers, individuals with medical conditions or mental disorders), which could compromise comparability across studies. No restrictions were imposed regarding date, type or status of publication, language, or study design. No formal cutoff for the sex distribution of the sample was predefined. During the final full-text assessment, 14 studies conducted during the early stages of the COVID-19 pandemic were excluded because they relied on non-probability online recruitment procedures and raised substantial concerns about sampling bias and limited representativeness of the Spanish general population (see Figure 1). These studies had markedly imbalanced samples, including an overrepresentation of women (>60 % of participants), young adults, highly educated participants and/or specific geographical regions (see Supplementary Table S1).

### Information sources

14 databases were consulted, selected for their complementary coverage, to retrieve relevant articles (see Table S2 in the Supplementary Material): MEDLINE (PubMed), Embase (Elsevier), all databases within the Web of Science (WoS) Core Collection (excluding Current Chemical Reactions and Index Chemicus), Scopus, PsycINFO (ProQuest), ÍnDICEs CSIC, and PSICODOC (EBSCOhost). The search and download of bibliographic records were conducted by one researcher (S.R.S.) in May 2024, and subsequently updated in October 2024 by another researcher (A.G.T.), independently and following the same methodology. In addition, the reference lists of the included studies were manually reviewed.

### Search strategy

The search strategy for each database was constructed using terms related to three key concepts: Spain, epidemiology, and depression. To identify terms related to epidemiology and depression, various sources were consulted. First, diagnostic categories and related terms from all editions of the DSM, as well as from the sixth (the first to include mental disorders) to the eleventh edition of the ICD, were used. Both manuals served to enhance the sensitivity of the search, given that the construct of “depression” is heterogeneous and has evolved over time. In addition, the Medical Subject Headings (MeSH), Emtree, and Psychological Index Terms thesauri were reviewed, along with the terms used in previous reviews on the global prevalence of depression [37], [42], [43], [44], [45]. Finally, the selected terms were searched within the title, abstract, descriptors/keywords, or subject fields of each database. The search strategy for each database is detailed in the Supplementary Material (see Tables S3–S13).

### Study selection process

After the search, duplicate records were removed, and two researchers (S.R.S. and A.G.T.) independently assessed the relevance of the remaining records based on their titles and abstracts. Subsequently, the reports deemed potentially relevant were retrieved and evaluated in full text by the same researchers. Discrepancies in both phases were resolved through consultation with a third researcher (E.N.). In cases where multiple reports corresponding to the same study were identified, only the one providing the most complete and relevant information for the aims of the review was selected.

### Data extraction process

Two researchers (S.R.S. and A.G.T.) analysed the aspects listed in Table 1 for each study. A third researcher (E.N.) reviewed the procedure to assess consistency between the two and to resolve any discrepancies. If an article did not provide all relevant data, additional information was requested from the corresponding author.

**Table 1:** Variables analysed for each study included in the systematic review.

Variable	Description
Reference	Author(s) and year of publication
Location	Place where the study was conducted
Period	Period or year in which the study was carried out in Spain
Data source	Name of the study, if available
Design	Type of study design
Age	Age range of participants (e.g., >18 years)
Mean age, SD or SE	Mean age of the sample (with standard deviation or standard error)
Sample size, %	Sample size (with percentage of women)
Response rate, %	Response rate expressed as a percentage (%)
Assessment method (cut-off)	Assessment method used (with cut-off score applied)
Prevalence of depression, % (95 % CI, SD or SE)	Proportion of individuals meeting criteria for major depressive disorder (MDD), persistent depressive disorder (PDD), or unspecified depression (UD), based on the assessment method used in each study. Data were extracted on the time frame measured (e.g., 12-month prevalence), as well as any accompanying statistical measures (95 % CI, SD or SE)
Quality assessment	Assessment of study quality using the Joanna Briggs Institute (JBI) tools
Other outcomes	– Mean score on the psychometric instrument used. – Prevalence of other diagnostic categories (e.g., adjustment disorder with depressed mood). – Incidence

### Risk of bias and quality assessment of individual studies

The quality of the included studies was assessed using the Joanna Briggs Institute (JBI) tools for prevalence studies [46] or cohort studies [47], as appropriate according to each study’s design. Each item in the JBI tool is related to a specific type of risk of bias, and a study was considered to be of high quality if it met at least 80 % of the items. Two researchers (S.R.S. and A.G.T.) independently carried out the assessment of each study, and discrepancies were resolved through consultation with a third researcher (E.N.).

### Synthesis methods

Due to the high degree of heterogeneity across the included studies, a quantitative meta-analysis was not feasible. The sources of heterogeneity were not limited to the use of different assessment instruments, but also included: (I) heterogeneous prevalence time frames (e.g., lifetime vs. 2-week); (II) wide temporal dispersion of the studies, conducted in different years and socioeconomic contexts; (III) mixed diagnostic approaches (screening tools vs. structured clinical interviews); (IV) variation in sampling strategies and study design; and (V) inconsistent reporting of key statistical parameters required for pooling (e.g., standard errors, confidence intervals). Alternative approaches such as random-effects pooling, meta-regression, or standardisation procedures were considered but deemed inappropriate, as they would have produced artificially homogenised and potentially misleading summary estimates [48], [49], [50]. For these reasons, a structured narrative synthesis was conducted, grouping studies by assessment instrument and prevalence time frame to maximise comparability. Comparisons were made only within instrument groups. No direct statistical comparison was attempted across different assessment tools, as this would conflate methodological artefacts with true variation.

## Results

### Study selection process

The initial search identified a total of 6,638 records. After removing 2,522 duplicates, 4,116 records remained for the screening phase. Following the examination of titles and abstracts, 3,956 records were excluded, resulting in 160 potentially eligible reports. These were assessed in full text, and 147 were excluded for various reasons. Ultimately, 13 studies were included in the review (see Figure 1).

The most frequent reason for exclusion (n=91) was that the reports belonged to the same study, as large-scale investigations such as the European Study of the Epidemiology of Mental Disorders (ESEMeD) often generated multiple publications. In addition, among studies conducted during the early phases of the COVID-19 pandemic, a recurring reason for exclusion (n=14) was the use of non-probability online recruitment procedures, which raised concerns about sampling bias, demographic imbalance, and limited representativeness of the Spanish general population.

### Quality assessment of individual studies

The majority of the studies were of high quality (n=11; 84.61 %), while the remaining two were of moderate quality. The lowest scoring item on the JBI tool was related to the assessment methods used. Although the use of psychometric instruments to estimate the prevalence of depression is widespread in research studies, it has been criticised for its potential to introduce classification bias [51], 52]. For this reason, when evaluating the validity of the assessment method, only studies that employed a diagnostic interview were considered to meet the criteria. The full quality assessment of the studies is presented in Supplementary Tables S14 and S15.

### Characteristics of the included studies

Table 2 lists the main characteristics of the studies included in the review. In total, 13 were retrieved. Of these, 1 formed part of an international study (7.7 %), 6 were European studies (46.15 %), 3 had national coverage (23.07 %), and 3 had regional coverage (23.07 %). The earliest study was conducted in 1995, and the most recent in 2021. In terms of study design, 11 were cross-sectional (84.61 %) and 2 were cohort studies (15.39 %). 8 were conducted face-to-face (61.53 %), 3 online (23.07 %), 1 by telephone (7.7 %), and 1 used a mixed design with a face-to-face first phase and a telephone-based second phase (7.7 %). Most studies were conducted in populations aged ≥18 years (84.61 %), except for 2 studies which included individuals aged ≥15 years (15.4 %). The smallest sample comprised 1,011 participants, while the largest included 21,546. The lowest response rate was 57.1 %, and the highest was 99.6 %. With regard to assessment methods, 5 studies used the Composite International Diagnostic Interview (CIDI) (38.46 %), 3 used the Patient Health Questionnaire (PHQ) (23.07 %), 2 the Mini-International Neuropsychiatric Interview (MINI) (15.4 %), 1 the Centre for Epidemiological Studies Depression Scale (CES-D 8) (7.7 %), 1 the Depression, Anxiety, and Stress Scale (DASS-21) (7.7 %), and 1 used the Beck Depression Inventory (BDI) in the first phase and the Schedules for Clinical Assessment in Neuropsychiatry (SCAN) in the second phase (7.7 %).

**Table 2:** Characteristics of the included studies.

Reference	Location^a^	Study period	Data source^b^	Design	Sample size (%♀)^c^	Response rate, %	Age range	Mean age (SD or SE)	Assessment method (cut-off score)	Quality (JBI)
Arias-de la Torre et al. [60]	Spain	2014/15	EESE	Cross-sectional *(Ftf)*	21,546 (50.8)	–	>18	–	PHQ-8 (≥10)	High
Ayuso-Mateos et al. [63]	Santander	1996/98	ODIN	Cross-sectional *(Ftf)*	1,250 (50)	I:90 II:83	18–64	–	I: BDI (>12) II: SCAN 2.0	High
Ayuso-Mateos et al. [56]	Madrid Barcelona	T1: 2020 T0: 2019/20	–	Cohort (T0:*Ftf*, T1:*Tel*)	T1:1,103 T0:1,881 (60.3)	68	>18	T1:55.29 (16.3) T0:54.82 (16.3)	Screening CIDI 3.0	High
Castellvi et al. [57]	Spain	T1: 2020 T0: 2019	–	Cohort (online)	T1:1,357 (56) T0:2,005 (51.5)	T1:99.48 T0:99.65	≥18	T1:50.86 (15.5) T0:49.92 (15.7)	Screening CIDI 3.0	High
Chaves et al. [64]	Spain	R6:2012/13 R3:2006/07	ESS	Cross-sectional *(Ftf)*	R6:1,889 (51.1) R3:1,877 (50.8)	R6:70.3 R3:65.9	≥15	R6:47 (18.0) R3:46 (18.9)	CES-D 8	High
Freeman et al. [55]	Spain	2011/12	COURAGE	Cross-sectional *(Ftf)*	4,583 (51.15)	69.9	≥18	48 (±0.33)	CIDI 3.0	High
Gabarrell-Pascuet et al. [62]	Spain	2021	MIND/COVID	Cross-sectional *(Tel)*	2,000 (51.5)	57.1	≥18	49.7 (16.4)	PHQ-8 (≥10)	Moderate
Gabilondo et al. [53]	Spain	2001/02	ESEMeD	Cross-sectional *(Ftf)*	5,473 (51.8)	78.6	≥18	46.7 (±0.2)	CIDI 3.0	High
Lépine et al. [58]	Spain	1995	DEPRES I	Cross-sectional *(Ftf)*	16,132 (51.6)	–	≥15	–	MINI (modified)	High
Navarro-Mateu et al. [54]	Murcia	2010/12	PEGASUS	Cross-sectional *(Ftf)*	2,621 (54.5)	67.4	≥18	48.6	CIDI 3.0	High
Porras-Segovia [59]	Andalusia	2013/14	PISMA-ep study	Cross-sectional *(Ftf)*	4,507 (50.9)	82	18–75	42.8	MINI 5.0	High
Valiente et al. [61]	Spain	2020	–	Cross-sectional (online)	1,951 (47.1)	–	18–75	45.16 (12.7)	PHQ-9 (≥10)	High
Velten et al. [65]	Spain	2020	–	Cross-sectional (online)	1,011 (51.5)	–	≥18	–	DASS-21	Moderate

COURAGE, Collaborative Research on Ageing in Europe; PEGASUS, Psychiatric Enquiry of a General populAtion of SoUtheast Spain; ESEMeD, European Study of the Epidemiology of Mental Disorders; PISMA-ep study, Plan Integral de Salud Mental de Andalucía; DEPRES, Depression Research in European Society; ODIN, European Outcome of Depression International Network; EESE, Encuesta Europea de Salud en España; ESS, European Social Survey; CIDI, Composite International Diagnostic Interview; MINI, Mini-International Neuropsychiatric Interview; BDI, Beck Depression Inventory; SCAN, Schedules for Clinical Assessment in Neuropsychiatry; PHQ, Patient Health Questionnaire; CES-D, Centre for Epidemiologic Studies Depression Scale; DASS, Depression, Anxiety and Stress Scale; R3/R6, ESS round; Ftf, face-to-face; Tel: telephone; ^a^If the study is international and includes a Spanish subsample, only “Spain” is shown. ^b^‘–’ indicates an individual study specifically designed for this purpose (ad hoc). ^c^If the study is international, only the Spanish subsample is reported.

### Prevalence of depression in Spain

The prevalence of depression across all studies ranged from 1.8 % to 36.5 %, depending on the assessment method, study period, and the prevalence time frame, with 4–22.1 % being the most representative range overall for MDD. Table 3 presents the main findings of the included studies, grouped by assessment method and study period to enhance comparability.

**Table 3:** Main findings of the included studies.

Assessment method	Reference	Study period	Sample size	Prevalence (%) (95 % CI, SD or SE)										Other findings
				Life		12 months		6 month	1 month	2 weeks		1 week	Other	
				MDD	PDD	MDD	PDD	MDD	MDD	MDD	PDD	Depression	MDD	
CIDI 3.0	Freeman et al. [55]	2011/12	4,583	–	–	9	–	–	–	–	–	–	–	
	Navarro-Mateu et al. [54]	2010/12	2,621	13.8 (12.1;15.6)	0.6 (0.3;1.5)	6 (5.1;7.0)	0.5 (0.2;1.4)	–	–	–	–	–	–	
	Gabilondo et al. [53]	2001/02	5,473	10.6 (0.5)	–	4 (0.3)	–	–	–	–	–	–	–	
Screening CIDI 3.0	Castellvi et al. [57]	T1:2020 T0:2019	T1:1,357 T0:2,005	–	–	6.5 (T0)	–	–	–	–	–	–	8.8 (T1)^a^	p (T0 vs. T1)=0.036 Incidence=5.3 %
	Ayuso-Mateos et al. [56]	T1:2020 T0:2019/20	T1:1,103 T0:1,881	–	–	3.06 (T0) (0.45)	–	–	12.0 (T1) (1.45)	–	–	–	–	p (T0 vs. T1)<0.001 Incidence=6.87 %
MINI	Porras-Segovia [59]	2013/14	4,507	–	–	–	–	–	–	6.5 (6.0;7.0)	–	–	–	
	Lépine et al. [58] ^b^	1995	16,132	–	–	–	–	6.2	–	–	–	–	–	
PHQ (≥10)	Gabarrell-Pascuet et al. [62]	2021	2,000	–	–	–	–	–	–	12.4	–	–	–	Mean score PHQ-8 (SD): 4.3(4.7)
	Valiente et al. [61]	2020	1,951	–	–	–	–	–	–	22.1 (20.1;24.0)	–	–	–	Mean score PHQ-9 (SD): 6.5(5.65)
	Arias-de la Torre et al. [60]	2014/15	21,546	–	–	–	–	–	–	6.18	–	–	–	
I: BDI (>12) II: SCAN 2.0	Ayuso-Mateos et al. [63]	1996/98	1,250	–	–	–	–	–	–	1.8 (1.1;3.0)	0.5 (0.2;1.2)	–	–	Prevalence of adjustment disorder with depressed mood: 0.2 %
CES-D 8	Chaves et al. [64]	R6:2012/13 R3:2006/07	R6:1,889 R3:1,877	–	–	–	–	–	–	–	–	R6:19.9^c^ R3:15.6^c^	–	Mean score CES-D 8 (SD) – R6: 6.07 (4.41) – R3: 5.60 (4.23)
DASS-21	Velten et al. [65]	2020	1,011	–	–	–	–	–	–	–	–	36.5^d^	–	Mean score DASS-21 (SD): 5.67(5.40)

MDD, major depressive disorder; PDD, persistent depressive disorder or dysthymia; R3/R6, ESS round; ^a^Prevalence since the start of confinement in Spain (14 March 2020). ^b^Used a modified version of the MINI to measure 6-months prevalence. ^c^Using a cut-off ≥10. ^d^Using a cut-off ≥7 on depression subscale.

The studies that used the CIDI showed an increase in the prevalence of depression in recent years. Regarding 12-month prevalence of MDD, Gabilondo et al. [53] reported a rate of 4 % for the period 2001–2002. Subsequently, Navarro-Mateu et al. [54] reported a prevalence of 6 % in 2010–2012, and Freeman et al. [55] reported 9 % for 2011–2012. As for lifetime prevalence of MDD, Gabilondo et al. [53] reported 10.6 % in 2001–2002, and later, Navarro-Mateu et al. [54] found a rate of 13.8 % for 2010–2012. In the cohort studies, Ayuso-Mateos et al. [56] found a 12-month prevalence of depression of 3.06 % at T0 (June 2019 – March 2020) and a one-month prevalence of 12 % at T1 (May – June 2020). Similarly, Castellvi et al. [57] reported a 12-month prevalence of 6.5 % at T0 (October – November 2019) and a prevalence of 8.8 % at T1 (November – December 2020), measured from the beginning of the lockdown in Spain (14 March 2020).

Regarding the studies that used the MINI, Lépine et al. [58] reported a 6-month prevalence of MDD of 6.2 % in 1995, while Porras-Segovia [59] found a 2-week prevalence of 6.5 % in 2013–2014.

With respect to the studies that used the PHQ to estimate the 2-week prevalence of MDD, Arias-de la Torre et al. [60] reported a prevalence of 8 % in women and 4.1 % in men for the period 2014–2015. Later, Valiente et al. [61] reported a prevalence of 22.1 % in 2020, with a mean PHQ-9 score of 6.5, while Gabarrell-Pascuet et al. [62] estimated a prevalence of 12.4 % in 2021, with a mean score of 4.3.

Using the BDI in the first phase (and the SCAN in the second), Ayuso-Mateos et al. [63] reported prevalence rates of 1.8 % for MDD, 0.5 % for PDD, and 0.2 % for adjustment disorder with depressed mood, for the period 1996–1998. In another study, Chaves et al. [64] used the CES-D 8 and reported a one-week prevalence of 15.6 %, with a mean score of 5.6 in 2006–2007, whereas in 2012–2013, the prevalence increased to 19.9 %, with a mean score of 6.07. Finally, Velten et al. [65] estimated a one-week prevalence of 36.5 % in 2020 using the DASS-21, with a mean score of 5.67.

### Variables associated with depression

All studies included in the review that reported gender-disaggregated estimates consistently showed that the prevalence of depression in women was twice as high as in men [53], [54], [55], [56, [58], [59], [60, 63]. In addition, studies that examined associations between depression and other variables found that being female, along with unfavourable sociodemographic and health-related conditions, was always associated with a significantly increased risk of depression [53], [54], [55], [56, 59], 60], 62]. These variables included: gender (female); marital status (single, separated, divorced, or widowed); low educational attainment; employment status (unemployed, disabled, early retired or retired, low-skilled jobs, jobs in the primary sector, or housework); low household income; medical conditions (e.g. cancer, obesity, cardiovascular disease); psychological factors (e.g. stressful life events, neuroticism); and loneliness during the early phase of the COVID-19 pandemic. Conversely, higher socioeconomic status, more years of education, physical activity, and resilience during the COVID-19 period were significantly associated with a lower risk of depression.

## Discussion

### Prevalence of depression in Spain

Among the studies included in the review that were methodologically comparable over time (11 of the 13 studies), all showed an increase in depression prevalence estimates, suggesting an increase in reported depression prevalence in Spain over recent decades [53], [54], [55], [56], [57], [58], [59], [60], [61], [62, 64]. Although the two studies that used the MINI report similar figures across different periods, Lépine et al. [58] used a modified version to measure 6-month prevalence, whereas Porras-Segovia [59] assessed 2-week prevalence. Given that MDD typically persists for months and tends to be recurrent, estimates of 6-month prevalence generally yield higher figures than 2-week prevalence. Therefore, greater differences between the findings of these two studies would be expected, and the same applies to the cohort studies, which measured prevalence using a broader time frame at T0 and a narrower range at T1 [56], 57].

**Figure 2:**
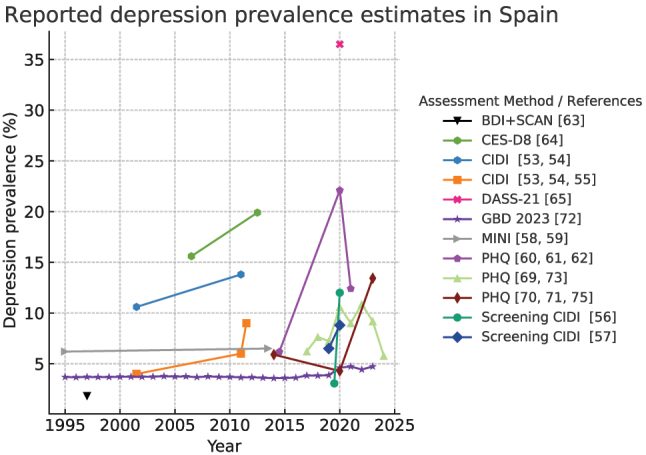
Reported depression prevalence estimates in Spain. All studies that are comparable in terms of assessment method and prevalence time frame show an increase in the estimated prevalence figures, with the exception of the values reported in the 2024 ESCA (see “Prevalence of Depression in Spain”). Direct comparison across studies should be avoided, as they employ different methodologies (see Tables 2 and 3). Studies [58], 59] and [56], 57] initially measured a wider prevalence time frame and later a narrower one. Therefore, the expected change would be greater than that reflected in their figures. CIDI: Composite International Diagnostic Interview. MINI: Mini-International Neuropsychiatric Interview. BDI: Beck Depression Inventory. SCAN: Schedules for Clinical Assessment in Neuropsychiatry. PHQ: Patient Health Questionnaire. CES-D: Centre for Epidemiologic Studies Depression Scale. DASS: Depression, Anxiety and Stress Scale. GBD 2023: Global Burden of Disease Study 2023.

The findings of the included studies are consistent with those reported in successive editions of the Spanish National Health Survey (ENSE) and the European Health Survey in Spain (EESE) – recently integrated into the Spanish Health Survey (ESdE) – as well as with the Catalan Health Survey (ESCA). All of these surveys are based on representative samples of the general population and assess depression in participants aged ≥15 years using the PHQ [66], [67], [68]. In the case of the ESCA, a rising trend can be observed from 6.2 % in 2017, 7.6 % in 2018, 7.2 % in 2019, 10.6 % in 2020, 9 % in 2021, 10.9 % in 2022, and 9.2 % in 2023 [69]. A similar upward pattern can also be noted when comparing data from the 2014 EESE, the 2023 ESdE, and the studies included in this review that used the PHQ. In the study by Arias-de la Torre et al. [60], which reports adjusted data from the 2014 EESE, the weighted prevalence was 6.18 %, while the unadjusted figure for the same survey was 5.9 % [70]. Subsequently, prevalence figures were 22.1 % in 2020 [61], 12.4 % in 2021 [62], and 13.43 % in the 2023 ESdE [71]. Lastly, these trends are similar to those reported in the GBD 2023 [72], which shows an increase in the estimated prevalence of depression in Spain from 2015 onwards (see Figure 2).

Two exceptions to these patterns are observed in the 2024 ESCA and the 2020 EESE. On the one hand, the prevalence of depression in the 2024 ESCA was 5.8 % [73], representing the lowest value in the entire series [69]. In this edition, the new company responsible for fieldwork was unable to begin data collection until April, resulting in the absence of information for the first quarter. Likewise, it is noteworthy that several sociodemographic variables differed markedly between the 2023 and 2024 surveys: *retired due to age*, 0.6 % vs. 12.9 %; *duration of unemployment >4 years* (within the unemployed population), 12.7 % vs. 95.4 %; and *previously employed in paid work*, 90.6 % vs. 49.1 % [73], 74]. On the other hand, the 2020 EESE reported an unadjusted prevalence of 4.28 % [75], representing a decrease compared to the 2014 EESE, where prevalence ranged between 6.18 % [60] and 5.9 % [70]. This discrepancy is likely explained by the shift from face-to-face to telephone interviews during the COVID-19 pandemic, which resulted in a significant increase in the percentage of inaccessible households (from 0.31 % to 25.4 %). In comparison, the proportion of inaccessible households before the pandemic was so low (0.15 % in the 2017 ENSE and 0.25 % in the 2014 EESE) that it “warranted no comment” in the analysis of nonresponse [76], [77], [78]. In light of these considerations, the results of the 2024 ESCA and the 2020 EESE should be interpreted with caution and in the context of previous and subsequent series.

Finally, it is important to note that the studies included in this review reveal a clear gender bias and a consistent association between depression and unfavourable sociodemographic conditions, a finding commonly reported in related research [6], 7]. In the 2023 ESCA, the prevalence of depression was twice as high in women as in men, with one in five women (20.4 %) reporting having experienced depression at some point in their lives [74]. Similarly, higher rates of depression were observed among individuals from lower social classes and among those with no education or only primary education [79]. According to the 2017 ENSE, the prevalence of depression was more than twice as high among women and the unemployed, 10 times higher among those unable to work, and showed a clear social class gradient [80]. In high-income countries analysed in the World Mental Health Survey (WMH Survey), MDD was significantly associated with being female, marital status (separated, divorced, widowed, or never married), living alone, and having low or lower-middle income levels [81].

### Comparison of depression prevalence in Spain with other countries

In studies conducted up to the early 2000s, Spain consistently ranked among the countries with the lowest prevalence rates. Thus, in the study by Lépine et al. [58], the 6-month prevalence of MDD in Spain (6.2 %) was lower than both the European mean and median (M=6.9 %, Med=6.5 %). Similarly, in the study by Ayuso-Mateos et al. [63], the lowest prevalence of MDD was found in Spain (1.8 %), notably lower than the European values (M=6.7 %, Med=6.1 %). Finally, both Bromet et al. [81] and Gabilondo et al. [53] drew on data from the ESEMeD study, later incorporated into the WMH Survey. Bromet et al. [81] compared the prevalence of MDD across 18 countries, and Spain reported a 12-month prevalence of 4 %, significantly lower than the figures among high-income countries (M=5.5 %, Med=4.95 %).

Later, drawing on data from the European Social Survey (ESS) conducted between 2006 and 2014, Spain moved to an intermediate position in terms of prevalence, showing early signs of increase compared with other countries. Beller et al. [82], Chaves et al. [64], and Dudal and Bracke [83] used data from the ESS, which, through the CES-D 8, provides information on depressive symptomatology from rounds three (R3: 2006), six (R6: 2012), and seven (R7: 2014). Based on data from R3 and R6, Dudal and Bracke [83] reported an estimated prevalence of depression in Spain of 20.78 % in women and 13.97 % in men, placing these figures around the European averages (M♀=18.77, M♂=13.83) and medians (Med♀=18, Med♂=12.5). Subsequently, Beller et al. [82] conducted a study using data from ESS rounds R3 and R7 and found a significant increase in depressive symptoms only among young women (aged 25–39) within the Spanish sample. In most of the 18 countries included, the observed changes were not statistically significant, but those that were showed a decrease in depressive symptoms, in contrast to what was observed in Spain.

Finally, in more recent studies, Spain has shown higher prevalence rates compared with other countries. In the study by Freeman et al. [55], Spain reported the highest depression prevalence (9 %), more than double the figures recorded in Finland and Poland (M=5.67, Med=4). Similarly, Velten et al. [65] reported a mean DASS-21 score of 5.67 points in Spain, above both the international mean and median (M=5.27, Med=5.37). Likewise, in the 2014 European Health Interview Survey (EHIS), the prevalence of major depressive symptoms in Spain measured using the PHQ-8 (3 %), was higher than the European mean and median (M=2.61, Med=2.4) [84]. Lastly, the study by Valiente et al. [61] formed part of the COVID-19 Psychological Research Consortium (C19PRC), and Shevlin et al. [85] published a study comparing mean PHQ-9 scores across countries. In this study, mean scores in Spain (6.50) were above the international mean and median (M=6.08, Med=6.14), whereas those in Ireland (5.78) and the United Kingdom (5.37) were lower.

Taken together, when examining Spain’s position in terms of prevalence across international studies over time, it appears that the country initially ranked lower in earlier studies [58], 63], 81] and eventually reached higher positions in the most recent ones [55], 65], [82], [83], [84], [85]. This pattern may indicate an upward trend in reported depression prevalence estimates in Spain, which may be more pronounced than those observed in other countries. However, the limited availability of directly comparable data warrants caution when interpreting these comparisons.

These trends are consistent with those reported by Ge et al. [8], who, drawing on data from the GBD, compared patterns in the incidence and disease burden of MDD across 204 countries and territories between 1993 and 2017, using the Age-Standardized Annual Incidence (ASAI) and DALYs. Notably, among all the countries and regions analysed, Spain showed the largest increase in both the incidence and burden of MDD (ASAI +28.26 %, DALYs +29.20 %), followed by Mexico (ASAI +19.99 %, DALYs +20.81 %) and Malaysia. Spain’s figures contrast sharply with the global decline of 6.7 % in ASAI and 6.8 % in DALYs, and are in line with the increases reported in countries such as Germany (ASAI +14.77 %, DALYs +15.74 %) and the United States (ASAI +18.12 %, DALYs +17.36 %). Nevertheless, a recent review by Cosgrove et al. [86] raised concerns about the data underlying GBD estimates of MDD prevalence, particularly regarding the instruments used in individual studies and their potential for overestimation.

### Prevalence of depression in Spain during the initial phase of the COVID-19 pandemic

Following the outbreak of the COVID-19 pandemic, multiple studies seeking to assess its impact on the incidence of depression began to proliferate [33], 87]. However, the vast majority of these studies exhibit critical conceptual and methodological flaws.

From a conceptual standpoint, it is important to recall the distinction between clinically significant depression and psychological distress. The former is characterised by the presence of a defined set of symptoms of sufficient duration and functional impairment, which cannot be better explained by social or cultural factors [88], 89]. In contrast, psychological distress encompasses a variable range of emotional and cognitive phenomena that may represent an expected and adaptive reaction to different stressors. Under the exceptional circumstances experienced during the early stages of the pandemic, most of these studies [33], 87] tend to psychiatrize normal human reactions, as they use screening instruments to measure phenomena such as sadness, anxiety or stress, and label them as “depressive symptoms”, instead of interpreting them as expressions of psychological distress reactive to the situation. This represents a classification bias and leads to an overestimation of the figures reported in these studies. From a methodological standpoint, many of these studies are based on online surveys employing non-random sampling methods, which introduces selection bias by producing samples that are not representative of the general population, as well as coverage bias as a result of the digital divide [90], 91].

Our review included two cohort studies [56], 57] and three cross-sectional studies [61], 62], 65], which, despite sharing similar conceptual issues, are less affected by the methodological limitations mentioned above and provide more representative data on the general population. In these cohort studies, prevalence increased from T0 to T1, with Ayuso-Mateos et al. [56] reporting a fourfold rise. Likewise, cross-sectional national studies reported that prevalence tripled during the first wave [61] and doubled during the third wave [62], compared with previous studies [60], 70]. Although Valiente et al. [61] used the PHQ-9 instead of the PHQ-8, scores for both instruments are highly comparable according to a meta-analysis [92], which found a correlation of 0.996. Internationally, the scores reported for Spain by Velten et al. [65] in 2020 were higher than both the mean and median across the eight countries included in the analysis. Taken together, these findings are consistent with those reported in different editions of the ESCA and the GBD. The ESCA showed a marked increase in the prevalence of depression in 2020, followed by a decrease in 2021 and a further rise in 2022. Similarly, the GBD reported a pronounced increase in 2020, a slight increase in 2021, and a decrease in 2022 [69], 72].

Although these studies report notable increases in the estimated prevalence of depression during the initial phases of the COVID-19 pandemic, they still retain the conceptual limitations discussed above. Thus, it is more likely that most of the observed increases reflect a rise in reactive psychological distress rather than such a marked increase in depressive disorders.

### Contextual factors that may help interpret the observed trends

Although the studies included in the review do not allow causal inferences to be drawn regarding the drivers of the observed trends, it is relevant to highlight several features of the healthcare, socioeconomic, and cultural context that may help interpret the patterns observed in Spain.

At the healthcare level, Spain has for many years maintained public healthcare expenditure below the EU average, allocating 6.75 % of GDP to health in 2023, compared with 8.05 % in the EU [20]. This underfunding is partly a legacy of the 2008 global financial crisis, when Spain, unlike northern European countries, implemented severe austerity policies that reduced the capacity of the National Health System (NHS) to meet population needs [93], [94], [95], [96]. Subsequently, the COVID-19 pandemic had a particularly severe impact in Spain [97], [98], [99], [100], contributing to widespread healthcare system overload and to the reallocation of human and structural mental health resources to COVID-19-related activities [101], [102], [103], [104]. In this context, low numbers of psychiatrists within the NHS have been reported compared with the European average (13.27 vs. 19.40 per 100,000 inhabitants in 2023), together with a low ratio of clinical psychologists (approximately 6 per 100,000) [105], [106], [107]. In parallel, a marked and sustained increase in psychotropic medication use has been observed among the Spanish population in recent decades [10], [11], [12], [13], [14], a pattern that may indicate greater reliance on pharmacological treatment in comparison with countries that allocate more resources to psychosocial interventions [108], [109], [110]. Taken together, sustained underinvestment in mental health and service overload may hinder the appropriate detection and management of depression and contribute to chronicity and relapse, thereby helping to contextualise the prevalence patterns observed in Spain [111], [112], [113], [114], [115], [116].

At the economic level, Spain was exposed more intensely and for longer than most European countries to the effects of both the 2008 global financial crisis and the COVID-19 pandemic [21]. Although the contraction in GDP in 2009 was similar to the EU average, Spain’s recovery diverged markedly from that of most European economies, remaining stagnant until 2014. Subsequently, in 2020, Spain experienced the second largest fall in GDP in Europe (−10.9 %), far exceeding the average reduction in the EU (−5.6 %). In line with this, the available evidence indicates that deep and prolonged economic contractions of this kind are associated with an increased population risk of depression by intensifying psychological stress, financial hardship, job insecurity, and housing difficulties [23], 24], 28], particularly among children and young people [23], 117].

At the social level, Spain has historically maintained one of the highest unemployment rates in Europe [118]. The aftermath of the 2008 crisis left one in four members of the Spanish labour force unemployed in 2013 (26.1 %) [17]. Following the COVID-19 pandemic, Spain still had the second highest unemployment rate in the EU in 2024, almost twice the EU average (11.4 % vs. 5.9 %). In this regard, unemployment has been consistently associated with a higher risk of depression through the economic and social strain it entails [23], 24], 27], 29], especially among young people, individuals in low-skilled jobs, and in contexts of marked inequality [27], 119]. Likewise, for many years Spain has shown, compared with the European average, higher levels of inequality on the Gini index, lower public expenditure on social protection, and a higher proportion of the population at risk of poverty or social exclusion, with one in four people at risk in 2024 (25.8 %) [18], 19], 120]. These unfavourable social conditions have been associated with a higher risk of depression through increased psychological stress, the erosion of social capital, and social isolation [25], 26], 121], especially among women and minority groups such as migrants and LGBTQ+ populations [25].

At the cultural level, the Spanish population has shown a greater willingness to seek professional mental health support compared with other European countries, such as Italy, France and Germany [122], 123]. Likewise, greater literacy about depression has been documented in recent years [124], 125], alongside lower levels of stigma towards it, particularly among young people and those with higher educational attainment [126]. These factors may have contributed to a greater cultural normalisation of depression, facilitated self-identification, and reduced social barriers to seeking care, thereby making a larger number of cases more visible. In this regard, self-perceived depression in the general population increased from 5.8 % in the 2009 EESE [127] to 7.80 % in the 2023 ESdE [71], and from 8 % in the 2018 ESCA [128] to 11.1 % in the 2024 edition [73]. Finally, unlike the pattern observed among men, identification with traditional female gender roles has been linked to higher rates of help-seeking, diagnosis, and treatment [129], [130], [131], [132], which could influence differential epidemiological visibility by gender.

### Strengths and limitations

The evidence presented in this review should be interpreted in light of certain limitations. First, only 13 studies were included, prioritising quality over quantity, which limits data availability and temporal coverage. Second, up to seven different assessment instruments were used across studies, within a context of evolving depression nosology. These instruments vary in their sensitivity and specificity [133], 134], which limits comparability and constrains direct comparison of prevalence estimates across studies. Third, 11 studies employed a cross-sectional design, which precludes the estimation of incidence or causal inference. Fourth, studies that included only specific populations (e.g., older adults, workers) were excluded, which may affect the representativeness of these subgroups. Fifth, self-reported data are subject to recall bias, and selection bias (e.g., individuals with more severe symptoms or fear of stigma) may contribute to an underestimation of prevalence figures. Studies conducted during the initial phases of the COVID-19 pandemic exhibited selection and classification biases that may have overestimated prevalence. Sixth, because a meta-analysis was not feasible due to the high degree of heterogeneity between studies, the risk of publication bias could not be assessed quantitatively. Finally, although no changes were made once the review had been designed, the protocol was not registered, which may limit transparency.

Rather than establishing a single definitive national prevalence estimate, the main strength of this study is that it provides a comprehensive and up-to-date structured synthesis of available, methodologically heterogeneous general-population studies reporting depression prevalence estimates in Spain over recent decades, helping to explain why reported estimates differ. To our knowledge, this is the first systematic review to address this topic. It includes studies with appropriate geographical coverage, substantial sample sizes, balanced gender distribution, and age ranges representative of the general population. Moreover, the vast majority of studies (n=11; 84.61 %) achieved a high quality rating (JBI). Finally, four relevant studies conducted in the Spanish population were identified [57], 62], 64], 65], yet their data are not incorporated into the current GBD estimates for Spain [135]. Including these studies in future iterations could enhance the accuracy and reliability of the estimates.

## Conclusions

While acknowledging the substantial methodological heterogeneity across available studies, the synthesised evidence suggests that reported depression prevalence estimates increased among comparable studies of the Spanish general population between 1995 and 2021. Across all studies, the prevalence observed in women was consistently twice that of men. Moreover, being female and experiencing unfavourable sociodemographic conditions were significantly associated with a higher risk of developing depression. Although studies conducted during the initial phases of the COVID-19 pandemic show a notable peak in depression, it is likely that the increase they reflect is driven mainly by a rise in reactive psychological distress, rather than being attributable to such a marked increase in depressive disorders. Finally, the available evidence suggests that reported depression prevalence estimates in Spain may have increased more markedly than those observed in other countries in recent decades, although the limited availability of directly comparable data warrants caution when interpreting cross-country comparisons.

Taken together, these findings may help guide future research and inform appropriate health policies. Future research on the prevalence of depression in Spain could benefit from randomised sampling, a first screening phase using the PHQ, and a second diagnostic phase using the CIDI, combined with a face-to-face longitudinal design. This methodology could strengthen the representativeness of the sample, enhance study efficiency and quality, reduce the risk of overdiagnosis, and allow for comparison and harmonisation of results with major regional (ESCA), national (ENSE/ESdE), and European (EESE) surveys, as well as other relevant international studies [81], 136].

From a health policy perspective, and taking into account the contextual factors discussed above, strengthening public mental health resources and promoting evidence-based psychosocial interventions may be relevant. In addition, preventive policies aimed at improving the population’s socioeconomic conditions, together with psychoeducational programmes that help distinguish reactive psychological distress from clinical depression, may be particularly useful in contexts of crisis. Finally, these actions should be developed through a gender-sensitive lens and with specific attention to socially disadvantaged groups.

## Supplementary Material

Supplementary Material
