# Supplementary material for: Depression prevalence estimates in Spain (1995–2021): a systematic review
Source: Open Med (Wars). 2026 Aug 10;21(1):20261498. doi: 10.1515/med-2026-1498 (PMC13454903; doi:10.1515/med-2026-1498)
Supplement: Supplementary file 1 [file j_med-2026-1498_suppl_001.docx]

**SUPPLEMENTARY MATERIAL**

**Eligibility Criteria**

| **Table S1** | |
| --- | --- |
| **Excluded early-pandemic studies with non-probability sampling and representativeness concerns** | |
| 1 | del Río-Casanova, L., Sánchez-Martín, M., García-Dantas, A., González-Vázquez, A., & Justo, A. (2021). Psychological Responses According to Gender during the Early Stage of COVID-19 in Spain. *International journal of environmental research and public health, 18*(7), 3731. <https://doi.org/10.3390/ijerph18073731> |
| 2 | García-Álvarez, L., de la Fuente-Tomás, L., García-Portilla, M. P., Sáiz, P. A., Lacasa, C. M., Dal Santo, F., González-Blanco, L., Bobes-Bascarán, M. T., García, M. V., Vázquez, C. Á., Iglesias, Á. V., Cao, C. M., Fernández, A. G., Bascarán Fernández, M. T., Fernández, A. P., Revuelta, J. R., Zazo, E. S., Madera, P. Z., Álvarez, M. S., Sánchez, Á. P., … Bobes, J. (2020). Early psychological impact of the 2019 coronavirus disease (COVID-19) pandemic and lockdown in a large Spanish sample. *Journal of global health, 10*(2), 020505. <https://doi.org/10.7189/jogh.10.020505> |
| 3 | García-Dantas, A., Justo-Alonso, A., González-Vázquez, A., Sánchez-Martín, M., & del Río-Casanova, L. (2021). Immediate Psychological Response during the Very Early Stage of the Coronavirus Pandemic (COVID-19) in a Spanish Community Sample. *Actas Españolas De Psiquiatría, 49*(4), 180–193. Retrieved from <https://actaspsiquiatria.es/index.php/actas/article/view/289> |
| 4 | García-Fernández, L., Romero-Ferreiro, V., Padilla, S., David López-Roldán, P., Monzó-García, M., & Rodriguez-Jimenez, R. (2021). Gender differences in emotional response to the COVID-19 outbreak in Spain. *Brain and behavior, 11*(1), e01934. <https://doi.org/10.1002/brb3.1934> |
| 5 | Garrido-Cumbrera, M., González-Marín, A., Correa-Fernández, J., Braçe, O., & Foley, R. (2023). Can Views and Contact with Nature at Home Help Combat Anxiety and Depression during the Pandemic? Results of the GreenCOVID study. *Brain and behavior, 13*(3), e2875. <https://doi.org/10.1002/brb3.2875> |
| 6 | Jacques-Aviñó, C., López-Jiménez, T., Medina-Perucha, L., de Bont, J., Gonçalves, A. Q., Duarte-Salles, T., & Berenguera, A. (2020). Gender-based approach on the social impact and mental health in Spain during COVID-19 lockdown: a cross-sectional study. *BMJ open, 10*(11), e044617. <https://doi.org/10.1136/bmjopen-2020-044617> |
| 7 | Jané-Llopis, E., Anderson, P., Segura, L., Zabaleta, E., Muñoz, R., Ruiz, G., Rehm, J., Cabezas, C., & Colom, J. (2021). Mental ill-health during COVID-19 confinement. *BMC psychiatry, 21*(1), 194. <https://doi.org/10.1186/s12888-021-03191-5> |
| 8 | Moya-Lacasa, C., Álvarez-Vázquez, C. M., González-Blanco, L., Valtueña-García, M., Martín-Gil, E., Seijo-Zazo, E., … Bobes, J. (2021). Impact of the coronavirus outbreak on mental health in the different Spanish regions. *Actas Españolas De Psiquiatría, 49*(2), 64–70. Retrieved from <https://actaspsiquiatria.es/index.php/actas/article/view/662> |
| 9 | Muñoz-Navarro, R., Cano Vindel, A., Schmitz, F., Cabello, R., & Fernández-Berrocal, P. (2021). Emotional Disorders During the COVID-19 Outbreak in Spain: The Role of Sociodemographic Risk Factors and Cognitive Emotion Regulation Strategies. *Health education & behavior : the official publication of the Society for Public Health Education, 48*(4), 412–423. <https://doi.org/10.1177/10901981211014101> |
| 10 | Odriozola-González, P., Planchuelo-Gómez, Á., Irurtia, M. J., & de Luis-García, R. (2022). Psychological symptoms of the outbreak of the COVID-19 confinement in Spain. *Journal of health psychology, 27*(4), 825–835. <https://doi.org/10.1177/1359105320967086> |
| 11 | Papandreou, C., Arija, V., Aretouli, E., Tsilidis, K. K., & Bulló, M. (2020). Comparing eating behaviours, and symptoms of depression and anxiety between Spain and Greece during the COVID-19 outbreak: Cross-sectional analysis of two different confinement strategies. *European eating disorders review : the journal of the Eating Disorders Association, 28*(6), 836–846. <https://doi.org/10.1002/erv.2772> |
| 12 | Planchuelo-Gómez, Á., Odriozola-González, P., Irurtia, M. J., & de Luis-García, R. (2020). Longitudinal evaluation of the psychological impact of the COVID-19 crisis in Spain. *Journal of affective disorders, 277*, 842–849. <https://doi.org/10.1016/j.jad.2020.09.018> |
| 13 | Salas Quijada, C., López-Contreras, N., López-Jiménez, T., Medina-Perucha, L., León-Gómez, B. B., Peralta, A., Arteaga-Contreras, K. M., Berenguera, A., Queiroga Gonçalves, A., Horna-Campos, O. J., Mazzei, M., Anigstein, M. S., Ribeiro Barbosa, J., Bardales-Mendoza, O., Benach, J., Borges Machado, D., Torres Castillo, A. L., & Jacques-Aviñó, C. (2023). Social Inequalities in Mental Health and Self-Perceived Health in the First Wave of COVID-19 Lockdown in Latin America and Spain: Results of an Online Observational Study. *International journal of environmental research and public health, 20*(9), 5722. <https://doi.org/10.3390/ijerph20095722> |
| 14 | Suso-Ribera, C., & Martín-Brufau, R. (2020). How Much Support Is There for the Recommendations Made to the General Population during Confinement? A Study during the First Three Days of the COVID-19 Quarantine in Spain. *International journal of environmental research and public health, 17*(12), 4382. <https://doi.org/10.3390/ijerph17124382> |

**Sources of Information**

Fourteen databases were used as sources of information: MEDLINE (PubMed), Embase (Elsevier), Web of Science Core Collection —excluding Chemical Indexes— (Web of Science), Scopus, PsycINFO (ProQuest), ÍnDICEs CSIC (ÍnDICEs CSIC), and PSICODOC (EBSCOhost). The final search and data download from each database were conducted on 15 October 2024. The ÍnDICEs CSIC database primarily includes research articles published in scientific journals in Spanish, whereas the PSICODOC database focuses on scientific publications predominantly edited in Spain, Portugal, and Latin America. Table S2 presents the temporal coverage range of the databases. Throughout all stages of the review, corresponding authors or institutional contacts were approached when necessary to obtain or verify relevant information.

| **Table S2** | | |
| --- | --- | --- |
| **Sources of Information** | | |
| **Database** | **Interface/Platform** | **Coverage** |
| MEDLINE | PubMed | 1946-present |
| Embase | Elsevier | 1947-present |
| Science Citation Index Expanded (SCI-EXPANDED) | Web of Science | 1900-present |
| Social Sciences Citation Index (SSCI) | Web of Science | 1956-present |
| Arts & Humanities Citation Index (AHCI) | Web of Science | 1975-present |
| Conference Proceedings Citation Index–Science (CPCI-S) | Web of Science | 1990-present |
| Conference Proceedings Citation Index–Social Science & Humanities (CPCI-SSH) | Web of Science | 1990-present |
| Book Citation Index–Science (BKCI-S) | Web of Science | 2005-present |
| Book Citation Index–Social Sciences & Humanities (BKCI-SSH) | Web of Science | 2005-present |
| Emerging Sources Citation Index (ESCI) | Web of Science | 2005-present |
| Scopus | Elsevier | 1788-present |
| PsycINFO | ProQuest | 1806-present |
| ÍnDICEs CSIC | ÍnDICEs CSIC | 1970-2022 |
| PSICODOC | EBSCOhost | 1975-present |

**Search Strategy**

Tables S3 and S4 (English) and S5 and S6 (Spanish) present the search terms used. These tables also outline the generic search strategy, which was subsequently adapted for each individual database. Given that ÍnDICEs CSIC and PSICODOC primarily include scientific publications edited in Spanish, pilot searches demonstrated that the most effective way to maximise results was to combine key terms in both English and Spanish. The translation of keywords was based on the Health Sciences Descriptors (DeCS). If a keyword did not match any DeCS term, a literal translation was applied. To maximise retrieval, no restrictions were applied regarding publication date, publication type or status, language, or study design.

| **Table S3** | | | |
| --- | --- | --- | --- |
| **Terms used in search strategy (English)** | | | |
| Source | Concept | | |
|  | Depression | Spain | Epidemiology |
| DSM | *“Atypical depression”*  *“Depressive neurosis”*  *“Depressive reaction”*  *“Dysthymic disorder”*  *“Major affective disorder”*  *“Major depression”*  *“Major depressive disorder”*  *“Major depressive episode”*  *“Persistent depressive disorder”*  *“Unipolar depression”* | *—* | *—* |
| ICD | *“Depressive disorder”*  *“Depressive neurosis”*  *“Depressive personality”*  *“Depressive reaction”*  *“Dysthymia”*  *“Dysthymic disorder”*  *“Hypothymic personality”*  *“Major depressive disorder”*  *“Neurotic depression”*  *“Neurotic depressive reaction”*  *“Neurotic depressive state”*  *“Psychogenic depression”*  *“Reactive depression”*  *“Recurrent depressive disorder”* | *—* | *—* |
| Thesauri | *“Atypical depression”*^E^  *“Chronic depression”*^E^  *“Depression”*^M, E^  *“Depressive disorder, major”*^M^  *“Depressive disorder”*^M, E^  *“Dysthymia”*^E^  *“Dysthymic Disorder”*^M^  *“Major affective disorder”*^E^  *“Major depression”*^E, P^  *“Persistent depressive disorder”*^P^  *“Reactive depression”*^E^ | *Spain*^M,E^ | *Epidemiology*^M,E, P^  *Incidence*^M,E^  *Prevalence*^M,E^ |
| Previous related revisions | *“Depression”*  *“Depressive disorder”*  *“Major depression”*  *“Major depression disorder”*  *“Major depressive disorder”*  *“MDD”* | *Spain* | *Epidemiology*  *Incidence*  *Prevalence* |

Abbreviations: M: MeSH term. E: Entree term. P: Psychological Index Terms.

| **Table S4** | | |
| --- | --- | --- |
| **Generic search strategy (English)** | | |
|  | Concept | Key terms |
| #1 | Spain | *(Spain)* |
| #2 | Epidemiology | *(Epidemiology OR Incidence OR Prevalence)* |
| #3 | Depression | *(“Atypical Depression” OR “Chronic depression” OR “Depression” OR “Depressive disorder” OR “Depressive Disorder, Major” OR “Depressive neurosis” OR “Depressive personality” OR “Depressive reaction” OR “Dysthymia” OR “Dysthymic disorder” OR “Hypothymic personality” OR “Major affective disorder” OR “Major depression” OR “Major depression disorder” OR “Major depressive disorder” OR “Major Depressive Episode” OR “MDD” OR “Neurotic depression” OR “Neurotic depressive reaction” OR “Neurotic depressive state” OR “Persistent depressive disorder” OR “Psychogenic depression” OR “Reactive depression” OR “Recurrent depressive disorder” OR “Unipolar depression”)* |
| #4 |  | (#1) *AND* (#2) *AND* (#3) |

| **Table S5** | | | |
| --- | --- | --- | --- |
| **Terms used in search strategy (Spanish)** | | | |
| Source | Concept | | |
|  | Depression | Spain | Epidemiology |
| DSM | “Depresión atípica”  “Depresión mayor”  “Depresión unipolar”  “Episodio depresivo mayor”  “Neurosis depresiva”  “Reacción depresiva”  “Trastorno afectivo mayor”  “Trastorno depresivo mayor”  “Trastorno depresivo persistente”  “Trastorno distímico” | — | — |
| ICD | “Depresión neurótica”  “Depresión psicógena”  “Depresión reactiva”  “Distimia”  “Estado depresivo neurótico”  “Neurosis depresiva”  “Personalidad depresiva”  “Personalidad hipotímica”  “Reacción depresiva”  “Reacción depresiva neurótica”  “Trastorno depresivo”  “Trastorno depresivo mayor”  “Trastorno depresivo recurrente”  “Trastorno distímico” | — | — |
| Thesauri | “Depresión atípica”^E^  “Depresión crónica”^E^  “Depresión”^M, E^  “Trastorno depresivo, mayor”^M^  “Trastorno depresivo”^M, E^  “Distimia”^E^  “Trastorno distímico”^M^  “Trastorno afectivo mayor”^E^  “Depresión mayor”^E, P^  “Trastorno depresivo persistente”^P^  “Depresión reactiva”^E^ | España^M,E^ | Epidemiología^M,E, P^  Incidencia^M,E^  Prevalencia^M,E^ |
| Previous related revisions | “Depresión”  “Depresión mayor”  “TDM”  “Trastorno de depresión mayor”  “Trastorno depresivo”  “Trastorno depresivo mayor” | España | Epidemiología  Incidencia  Prevalencia |

Abbreviations: M: MeSH term. E: Entree term. P: Psychological Index Terms.

| **Table S6** | | |
| --- | --- | --- |
| **Generic search strategy (Spanish)** | | |
|  | Concept | Key terms |
| #1 | Spain | (España) |
| #2 | Epidemiology | (Epidemiología OR Incidencia OR Prevalencia) |
| #3 | Depression | (“Depresión” OR “Depresión atípica” OR “Depresión crónica” OR “Depresión mayor” OR “Depresión neurótica” OR “Depresión psicógena” OR “Depresión reactiva” OR “Depresión unipolar” OR “Distimia” OR “Episodio depresivo mayor” OR “Estado depresivo neurótico” OR “Neurosis depresiva” OR “Personalidad depresiva” OR “Personalidad hipotímica” OR “Reacción depresiva” OR “Reacción depresiva neurótica” OR “TDM” OR “Trastorno afectivo mayor” OR “Trastorno de depresión mayor” OR “Trastorno depresivo” OR “Trastorno depresivo mayor” OR “Trastorno depresivo persistente” OR “Trastorno depresivo recurrente” OR “Trastorno depresivo, mayor” OR “Trastorno distímico”) |
| #4 |  | (#1) AND (#2) AND (#3) |

PSICODOC does not include a thesaurus but provides a bibliographic search tool based on indexed terms, referred to as “subject terms”. Pilot searches revealed that using “subject terms” in an index-based search retrieved a specific but limited number of articles, which led to the decision to exclude this search method. The search strategy used for each database is detailed in Tables S7 to S13.

**MEDLINE (PubMed)**

| **Table S7** | |
| --- | --- |
| **Search strategy in MEDLINE** | |
| #1 | ("Spain"[Title/Abstract]) OR ("Spain"[MeSH Terms]) |
| #2 | ("Epidemiology"[Title/Abstract] OR "Incidence"[Title/Abstract] OR "Prevalence"[Title/Abstract] OR "Epidemiology"[Mesh] OR "Prevalence"[Mesh] OR "Incidence"[Mesh]) |
| #3 | (“Atypical Depression”[Title/Abstract] OR “Chronic depression”[Title/Abstract] OR “Depression”[Title/Abstract] OR “Depressive disorder”[Title/Abstract] OR “Depressive Disorder, Major”[Title/Abstract] OR “Depressive neurosis”[Title/Abstract] OR “Depressive personality”[Title/Abstract] OR “Depressive reaction”[Title/Abstract] OR “Dysthymia”[Title/Abstract] OR “Dysthymic disorder”[Title/Abstract] OR “Hypothymic personality”[Title/Abstract] OR “Major affective disorder”[Title/Abstract] OR “Major depression”[Title/Abstract] OR “Major depression disorder”[Title/Abstract] OR “Major depressive disorder”[Title/Abstract] OR “Major Depressive Episode”[Title/Abstract] OR “MDD”[Title/Abstract] OR “Neurotic depression”[Title/Abstract] OR “Neurotic depressive reaction”[Title/Abstract] OR “Neurotic depressive state”[Title/Abstract] OR “Persistent depressive disorder”[Title/Abstract] OR “Psychogenic depression”[Title/Abstract] OR “Reactive depression”[Title/Abstract] OR “Recurrent depressive disorder”[Title/Abstract] OR “Unipolar depression”[Title/Abstract] OR "Depression"[Mesh] OR "Depressive Disorder"[Mesh] OR "Dysthymic Disorder"[Mesh]) |
| #4 | #1 AND #2 AND #3 |

**Embase (Elseviere)**

| **Table S8** | |
| --- | --- |
| **Search strategy in Embase** | |
| #1 | ('spain':ab,ti OR 'spain'/exp) |
| #2 | ('epidemiology':ab,ti OR 'prevalence':ab,ti OR 'incidence':ab,ti OR 'epidemiology'/exp OR 'prevalence'/exp OR 'incidence'/exp) |
| #3 | ('atypical depression':ab,ti OR 'chronic depression':ab,ti OR 'depression':ab,ti OR 'depressive disorder':ab,ti OR 'depressive disorder, major':ab,ti OR 'depressive neurosis':ab,ti OR 'depressive personality':ab,ti OR 'depressive reaction':ab,ti OR 'dysthymia':ab,ti OR 'dysthymic disorder':ab,ti OR 'hypothymic personality':ab,ti OR 'major affective disorder':ab,ti OR 'major depression':ab,ti OR 'major depression disorder':ab,ti OR 'major depressive disorder':ab,ti OR 'major depressive episode':ab,ti OR 'mdd':ab,ti OR 'neurotic depression':ab,ti OR 'neurotic depressive reaction':ab,ti OR 'neurotic depressive state':ab,ti OR 'persistent depressive disorder':ab,ti OR 'psychogenic depression':ab,ti OR 'reactive depression':ab,ti OR 'recurrent depressive disorder':ab,ti OR 'unipolar depression':ab,ti OR 'atypical depression'/exp OR 'chronic depression'/exp OR 'depression'/exp OR 'depressive disorder'/exp OR 'dysthymia'/exp OR 'major affective disorder'/exp OR 'major depression'/exp OR 'reactive depression'/exp) |
| #4 | #1 AND #2 AND #3 |
| #5 | #1 AND #2 AND #3 AND ([embase]/lim OR [preprint]/lim)*** |

**Web of Science (WOS)**

| **Table S9** | |
| --- | --- |
| **Search strategy in WOS** | |
| Search in: Web of Science Core Collection  Editions:   - SCI-EXPANDED: Science Citation Index Expanded. - SSCI: Social Sciences Citation Index. - AHCI: Arts & Humanities Citation Index. - CPCI-S: Conference Proceedings Citation Index–Science. - CPCI-SSH: Conference Proceedings Citation Index–Social Science & Humanities. - BKCI-S: Book Citation Index–Science. - BKCI-SSH: Book Citation Index–Social Sciences & Humanities. - ESCI: Emerging Sources Citation Index. | |
| #1 | TS=(Spain) |
| #2 | TS=(Epidemiology OR Prevalence OR Incidence) |
| #3 | TS=(“Atypical Depression” OR “Chronic depression” OR “Depression” OR “Depressive disorder” OR “Depressive Disorder, Major” OR “Depressive neurosis” OR “Depressive personality” OR “Depressive reaction” OR “Dysthymia” OR “Dysthymic disorder” OR “Hypothymic personality” OR “Major affective disorder” OR “Major depression” OR “Major depression disorder” OR “Major depressive disorder” OR “Major Depressive Episode” OR “MDD” OR “Neurotic depression” OR “Neurotic depressive reaction” OR “Neurotic depressive state” OR “Persistent depressive disorder” OR “Psychogenic depression” OR “Reactive depression” OR “Recurrent depressive disorder” OR “Unipolar depression”) |
| #4 | #1 AND #2 AND #3 |

**Scopus**

| **Table S10** | |
| --- | --- |
| **Search strategy in Scopus** | |
| #1 | TITLE-ABS-KEY ( spain ) |
| #2 | TITLE-ABS-KEY ( epidemiology OR prevalence OR incidence) |
| #3 | TITLE-ABS-KEY ( "Atypical Depression" OR "Chronic depression" OR "Depression" OR "Depressive disorder" OR "Depressive Disorder, Major" OR "Depressive neurosis" OR "Depressive personality" OR "Depressive reaction" OR "Dysthymia" OR "Dysthymic disorder" OR "Hypothymic personality" OR "Major affective disorder" OR "Major depression" OR "Major depression disorder" OR "Major depressive disorder" OR "Major Depressive Episode" OR "MDD" OR "Neurotic depression" OR "Neurotic depressive reaction" OR "Neurotic depressive state" OR "Persistent depressive disorder" OR "Psychogenic depression" OR "Reactive depression" OR "Recurrent depressive disorder" OR "Unipolar depression" ) |
| #4 | #1 AND #2 AND #3 |

**PsycInfo (ProQuest)**

| **Table S11** | |
| --- | --- |
| **Search strategy in PsycInfo** | |
| S1 | tiab(Spain) |
| S2 | tiab(Epidemiology OR Prevalence OR Incidence) OR MAINSUBJECT.EXACT.EXPLODE("Epidemiology") |
| S3 | tiab("Atypical Depression" OR "Chronic depression" OR "Depression" OR "Depressive disorder" OR "Depressive Disorder, Major" OR "Depressive neurosis" OR "Depressive personality" OR "Depressive reaction" OR "Dysthymia" OR "Dysthymic disorder" OR "Hypothymic personality" OR "Major affective disorder" OR "Major depression" OR "Major depression disorder" OR "Major depressive disorder" OR "Major Depressive Episode" OR "MDD" OR "Neurotic depression" OR "Neurotic depressive reaction" OR "Neurotic depressive state" OR "Persistent depressive disorder" OR "Psychogenic depression" OR "Reactive depression" OR "Recurrent depressive disorder" OR "Unipolar depression") OR (MAINSUBJECT.EXACT.EXPLODE("Major Depression") OR MAINSUBJECT.EXACT.EXPLODE("Persistent Depressive Disorder")) |
| S4 | [S1] AND [S2] AND [S3] |

**ÍnDICEs CSIC (ÍnDICEs CSIC)**

| **Table S12** | |
| --- | --- |
| **Search strategy in ÍnDICEs CSIC** | |
| 1. | [Por Campos] Título documento(Alguna palabra)="España" O Resumen documento(Alguna palabra)="España" |
| 2. | [Por Campos] Título documento(Alguna palabra)="Epidemiología OR prevalencia OR incidencia" O Resumen documento(Alguna palabra)="Epidemiología OR prevalencia OR incidencia" |
| 3. | [Por Campos] Título documento(Alguna palabra)="(“Depresión” OR “Depresión atípica” OR “Depresión crónica” OR “Depresión mayor” OR “Depresión neurótica” OR “Depresión psicógena” OR “Depresión reactiva” OR “Depresión unipolar” OR “Distimia” OR “Episodio depresivo mayor” OR “Estado depresivo neurótico” OR “Neurosis depresiva” OR “Personalidad depresiva” OR “Personalidad hipotímica” OR “Reacción depresiva” OR “Reacción depresiva neurótica” OR “TDM” OR “Trastorno afectivo mayor” OR “Trastorno de depresión mayor” OR “Trastorno depresivo” OR “Trastorno depresivo mayor” OR “Trastorno depresivo persistente” OR “Trastorno depresivo recurrente” OR “Trastorno depresivo, mayor” OR “Trastorno distímico”)" O Resumen documento(Alguna palabra)="(“Depresión” OR “Depresión atípica” OR “Depresión crónica” OR “Depresión mayor” OR “Depresión neurótica” OR “Depresión psicógena” OR “Depresión reactiva” OR “Depresión unipolar” OR “Distimia” OR “Episodio depresivo mayor” OR “Estado depresivo neurótico” OR “Neurosis depresiva” OR “Personalidad depresiva” OR “Personalidad hipotímica” OR “Reacción depresiva” OR “Reacción depresiva neurótica” OR “TDM” OR “Trastorno afectivo mayor” OR “Trastorno de depresión mayor” OR “Trastorno depresivo” OR “Trastorno depresivo mayor” OR “Trastorno depresivo persistente” OR “Trastorno depresivo recurrente” OR “Trastorno depresivo, mayor” OR “Trastorno distímico”)" |
| 4. | [Combinada] ('1' AND '2' AND '3') |
| 5. | [Por Campos] Título documento(Alguna palabra)="Spain" O Resumen documento(Alguna palabra)="Spain" |
| 6. | [Por Campos] Título documento(Alguna palabra)="Epidemiology OR Incidence OR Prevalence" O Resumen documento(Alguna palabra)="Epidemiology OR Incidence OR Prevalence" |
| 7. | [Por Campos] Título documento(Alguna palabra)="(“Atypical Depression” OR “Chronic depression” OR “Depression” OR “Depressive disorder” OR “Depressive Disorder, Major” OR “Depressive neurosis” OR “Depressive personality” OR “Depressive reaction” OR “Dysthymia” OR “Dysthymic disorder” OR “Hypothymic personality” OR “Major affective disorder” OR “Major depression” OR “Major depression disorder” OR “Major depressive disorder” OR “Major Depressive Episode” OR “MDD” OR “Neurotic depression” OR “Neurotic depressive reaction” OR “Neurotic depressive state” OR “Persistent depressive disorder” OR “Psychogenic depression” OR “Reactive depression” OR “Recurrent depressive disorder” OR “Unipolar depression”)" O Resumendocumento(Alguna palabra)="(“Atypical Depression” OR “Chronic depression” OR “Depression” OR “Depressive disorder” OR “Depressive Disorder, Major” OR “Depressive neurosis” OR “Depressive personality” OR “Depressive reaction” OR “Dysthymia” OR “Dysthymic disorder” OR “Hypothymic personality” OR “Major affective disorder” OR “Major depression” OR “Major depression disorder” OR “Major depressive disorder” OR “Major Depressive Episode” OR “MDD” OR “Neurotic depression” OR “Neurotic depressive reaction” OR “Neurotic depressive state” OR “Persistent depressive disorder” OR “Psychogenic depression” OR “Reactive depression” OR “Recurrent depressive disorder” OR “Unipolar depression”)" |
| 8. | [Combinada] ('5' AND '6' AND '7') |

**PSICODOC**

| **Table S13** | |
| --- | --- |
| **Search strategy in PSICODOC** | |
| S1 | TI España OR AB España |
| S2 | TI ( Epidemiología OR Prevalencia OR Incidencia ) OR AB ( Epidemiología OR Prevalencia OR Incidencia ) |
| S3 | TI ( (“Depresión” OR “Depresión atípica” OR “Depresión crónica” OR “Depresión mayor” OR “Depresión neurótica” OR “Depresión psicógena” OR “Depresión reactiva” OR “Depresión unipolar” OR “Distimia” OR “Episodio depresivo mayor” OR “Estado depresivo neurótico” OR “Neurosis depresiva” OR “Personalidad depresiva” OR “Personalidad hipotímica” OR “Reacción depresiva” OR “Reacción depresiva neurótica” OR “TDM” OR “Trastorno afectivo mayor” OR “Trastorno de depresión mayor” OR “Trastorno depresivo” OR “Trastorno depresivo mayor” OR “Trastorno depresivo persistente” OR “Trastorno depresivo recurrente” OR “Trastorno depresivo, mayor” OR “Trastorno distímico”) ) OR AB ( (“Depresión” OR “Depresión atípica” OR “Depresión crónica” OR “Depresión mayor” OR “Depresión neurótica” OR “Depresión psicógena” OR “Depresión reactiva” OR “Depresión unipolar” OR “Distimia” OR “Episodio depresivo mayor” OR “Estado depresivo neurótico” OR “Neurosis depresiva” OR “Personalidad depresiva” OR “Personalidad hipotímica” OR “Reacción depresiva” OR “Reacción depresiva neurótica” OR “TDM” OR “Trastorno afectivo mayor” OR “Trastorno de depresión mayor” OR “Trastorno depresivo” OR “Trastorno depresivo mayor” OR “Trastorno depresivo persistente” OR “Trastorno depresivo recurrente” OR “Trastorno depresivo, mayor” OR “Trastorno distímico”) ) |
| S4 | S1 AND S2 AND S3 |
| S5 | TI Spain OR AB Spain |
| S6 | TI ( Epidemiology OR Prevalence OR Incidence ) OR AB ( Epidemiology OR Prevalence OR Incidence ) |
| S7 | TI ( (“Atypical Depression” OR “Chronic depression” OR “Depression” OR “Depressive disorder” OR “Depressive Disorder, Major” OR “Depressive neurosis” OR “Depressive personality” OR “Depressive reaction” OR “Dysthymia” OR “Dysthymic disorder” OR “Hypothymic personality” OR “Major affective disorder” OR “Major depression” OR “Major depression disorder” OR “Major depressive disorder” OR “Major Depressive Episode” OR “MDD” OR “Neurotic depression” OR “Neurotic depressive reaction” OR “Neurotic depressive state” OR “Persistent depressive disorder” OR “Psychogenic depression” OR “Reactive depression” OR “Recurrent depressive disorder” OR “Unipolar depression”) ) OR AB ( (“Atypical Depression” OR “Chronic depression” OR “Depression” OR “Depressive disorder” OR “Depressive Disorder, Major” OR “Depressive neurosis” OR “Depressive personality” OR “Depressive reaction” OR “Dysthymia” OR “Dysthymic disorder” OR “Hypothymic personality” OR “Major affective disorder” OR “Major depression” OR “Major depression disorder” OR “Major depressive disorder” OR “Major Depressive Episode” OR “MDD” OR “Neurotic depression” OR “Neurotic depressive reaction” OR “Neurotic depressive state” OR “Persistent depressive disorder” OR “Psychogenic depression” OR “Reactive depression” OR “Recurrent depressive disorder” OR “Unipolar depression”) ) |
| S8 | S5 AND S6 AND S7 |
| S9 | S4 OR S8 |

**Quality assessment**

| **Table S14** | | | | | | | | | | | |
| --- | --- | --- | --- | --- | --- | --- | --- | --- | --- | --- | --- |
| **JBI Critical Appraisal Checklist for Studies Reporting Prevalence Data** | | | | | | | | | | | |
|  | **Q1** | **Q2** | **Q3** | **Q4** | **Q5** | **Q6** | **Q7** | **Q8** | **Q9** | **Total** | **Quality** |
| Arias-de la Torre et al. [60] | Y | Y | Y | Y | Y | U | Y | Y | Y | 8 | High |
| Ayuso-Mateos et al. [63] | U | Y | Y | U | Y | Y | Y | Y | Y | 7 | High |
| Chaves et al. [64] | Y | Y | Y | Y | Y | U | Y | Y | Y | 8 | High |
| Freeman et al. [55] | Y | Y | Y | Y | Y | Y | Y | Y | Y | 9 | High |
| Gabarrell-Pascuet et al. [62] | Y | Y | Y | Y | U | U | Y | Y | U | 6 | Moderate |
| Gabilondo et al. [53] | Y | Y | Y | Y | Y | Y | Y | Y | Y | 9 | High |
| Lépine et al. [58] | Y | Y | Y | U | U | Y | Y | Y | Y | 7 | High |
| Navarro-Mateu et al. [54] | Y | Y | Y | Y | Y | Y | Y | Y | Y | 9 | High |
| Porras-Segovia [59] | Y | Y | Y | Y | Y | Y | Y | Y | Y | 9 | High |
| Valiente et al. [61] | Y | Y | Y | Y | Y | U | Y | Y | Y | 8 | High |
| Velten et al. [65] | Y | Y | Y | Y | U | U | Y | Y | U | 6 | Moderate |
| Total (%) | 90,9 | 100 | 100 | 81,81 | 72,72 | 54,54 | 100 | 100 | 81,81 |  |  |
| Questions:   - Q1 – Selection bias: Was the sample frame appropriate to address the target population? - Q2 – Selection bias: Were study participants recruited in an appropriate way? - Q3 – Selection bias/Statistical precision: Was the sample size adequate? - Q4 – Information bias/external validity: Were the study subjects and setting described in detail? - Q5 – Exclusion bias/non-response bias: Was data analysis conducted with sufficient coverage of the identified sample? - Q6 – Classification bias: Were valid methods used for the identification of the condition? - Q7 – Measurement bias: Was the condition measured in a standard, reliable way for all participants? - Q8 – Analytical bias: Was there appropriate statistical analysis? - Q9 – Non-response bias: Was the response rate adequate, and if not, was the low response rate managed appropriately? | | | | | | | | | | | |
| Quality   - ≥ 7 (≥80%)=High - ≥ 5 y <7 (≥50% y <80%)=Moderate - < 5 (<50%)=Low | | | | | | | | | | | |
| Abbreviations: Q: question. Y: yes. N: no. U: unclear. Note: each question is accompanied by the type of statistical bias to which it is related. | | | | | | | | | | | |

| **Table S15** | | | | | | | | | | | | | |
| --- | --- | --- | --- | --- | --- | --- | --- | --- | --- | --- | --- | --- | --- |
| **JBI critical appraisal checklist for cohort studies** | | | | | | | | | | | | | |
|  | **Q1** | **Q2** | **Q3** | **Q4** | **Q5** | **Q6** | **Q7** | **Q8** | **Q9** | **Q10** | **Q11** | **Total/11** | **Quality** |
| Ayuso-Mateos et al. [56] | Y | Y | Y | Y | Y | Y | U | Y | Y | U | Y | 9 | High |
| Castellvi et al. [57] | Y | Y | Y | Y | Y | Y | U | Y | U | Y | Y | 9 | High |
| Questions:   - Q1 – Selection bias: Were the two groups similar and recruited from the same population? - Q2 – Classification bias: Were the exposures measured similarly to assign people to both exposed and unexposed groups? - Q3 – Measurement bias: Was the exposure measured in a valid and reliable way? - Q4 – Confounding bias: Were confounding factors identified? - Q5 – Confounding bias: Were strategies to deal with confounding factors stated? - Q6 – Temporal bias: Were the groups/participants free of the outcome at the start of the study (or at the moment of exposure)? - Q7 – Classification bias: Were the outcomes measured in a valid and reliable way? - Q8 – Insufficient follow-up bias: Was the follow up time reported and sufficient to be long enough for outcomes to occur? - Q9 – Attrition bias: Was follow up complete, and if not, were the reasons to loss to follow up described and explored? - Q10 – Attrition bias: Were strategies to address incomplete follow up utilized? - Q11 – Analytical bias: Was appropriate statistical analysis used? | | | | | | | | | | | | | |
| Quality:   - ≥ 9 (80%)= High - ≥ 6 y <9 (≥50% y <80%)=Moderate - < 6 (<50%)=Low | | | | | | | | | | | | | |
| Abbreviations: Q: question. Y: yes. N: no. U: unclear. Note: each question is accompanied by the type of statistical bias to which it is related. | | | | | | | | | | | | | |
